# Large Anorectal Venous Malformation Treated With Microfoam Therapy

**DOI:** 10.14309/crj.0000000000001015

**Published:** 2023-03-31

**Authors:** Ashish Kumar Jha, Arya Suchismita, Vishwa Mohan Dayal

**Affiliations:** 1 Department of Gastroenterology, Indira Gandhi Institute of Medical Sciences, Sheikhpura, Patna, India

**Keywords:** anorectal venous malformation, endoscopic injection sclerotherapy, foam sclerotherapy

## Abstract

Deficiency of smooth muscle cells can lead to dysfunction and engorgement of blood vessels termed as hemangioma, arteriovenous malformations, and venous malformations (VMs). Anorectal VM is a rare disease. It can present with massive hematochezia. An optimal treatment of anorectal VMs has not been defined. Surgery is an option if the lesion can be resected completely. Endoscopic injection sclerotherapy has been reported to be effective in treating small colorectal VMs. However, it has rarely been described in the treatment of large VMs. In this study, we describe a rare case of large anorectal VMs treated with microfoam sclerotherapy.

## INTRODUCTION

Deficiency of smooth muscle cells can lead to engorgement of blood vessels termed as hemangioma, arteriovenous malformations, and venous malformations (VMs). VM occurs mainly in the alimentary tract, but it also affects the urogenital tract and other parts of the body. Multiple organ involvement is usually seen in Klippel-Trenaunay syndrome, blue rubber bleb nevus, and Osler-Weber-Rendu syndrome. VMs can cause serious problems due to acute or chronic bleeding, obstruction, and compression.^1^

Anorectal VM is a rare disease.^2^ Clinical features include minimal to massive hematochezia.^3,4^ Visualization of bluish red or red purple elevated lesions in colonoscopy is the diagnostic feature of VMs. The differential diagnosis includes hemangioma and arterial-VM. The absence of pulsation differentiates VMs and arteriovenous malformations. Biopsy is not recommended. Intravascular calcifications known as phleboliths are a specific finding of VMs seen on cross-sectional imaging. An optimal treatment of anorectal VMs has not been clearly defined.^2,3^ In this study, we describe a rare case of large anorectal VMs treated with microfoam sclerotherapy.

## CASE REPORT

**Figure 1.**
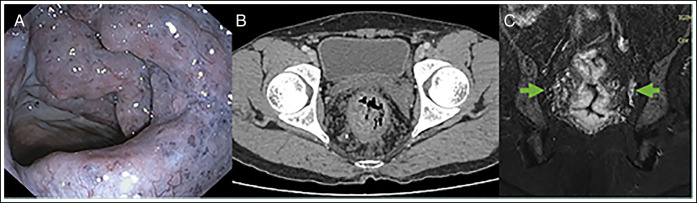
(A) Colonoscopy image showing bluish red elevated lesions occupying the complete circumference of the rectum. (B) Computed tomography scan showing diffuse thickening of the anorectal wall and phleboliths. (C) T2-weighted magnetic resonance imaging showing diffuse anorectal wall thickening and increased mesorectal vascularity (arrows).

A 42-year-old man presented with a 2-month history of hematochezia. He required 5 units of packed red blood cell transfusions. Examination showed pallor. Laboratory tests revealed a hemoglobin of 7.0 g/dL. Colonoscopy showed bluish red elevated vascular lesions occupying the complete circumference of the anorectum extending up to 14 cm of the anal verge (Figure 1). A computed tomography scan revealed diffuse thickening of the anorectal wall and phleboliths (Figure 1). T2-weighted (T2W) pelvic magnetic resonance imaging (MRI) showed diffuse anorectal wall thickening (Figure 1). Vascular lesions were also seen in the urinary bladder wall and mesorectal fat planes. Cystoscopy confirmed VMs. Vascular lesions were not present in the other organs, except the pelvic organs. The patient was diagnosed as having anorectal VMs with involvement of the mesorectum and urinary bladder. Packed red blood cells were transfused, and a laxative was started.

**Figure 2.**
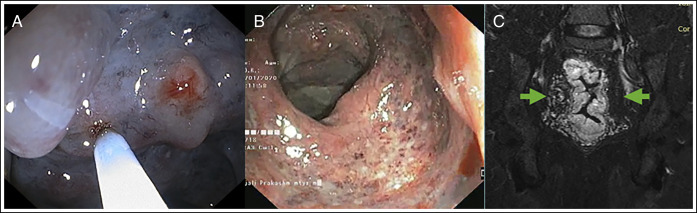
(A) Colonoscopy image showing injection microfoam sclerotherapy. (B) Follow-up colonoscopy image showing remarkable sclerosis of venous malformation. (C) T2-weighted magnetic resonance imaging showing decreased anorectal wall thickening and increased mesorectal fat signal (arrows).

Endoscopic foam sclerotherapy was planned after interdisciplinary team discussion. In the event of a complication or failure of sclerotherapy, options such as embolization of the superior rectal artery or surgery were considered. The foam was prepared by mixing the liquid polidocanol with CO_2_ with the help of disposable syringes, a CO_2_ gas connector, and a sclerotherapy needle (21-gauge) connected by a 3-way stopcock (modified Tessari technique). Two 20 mL plastic syringes were connected by a 3-way stopcock. The foam was formed by mixing polidocanol (1%) (Asklerol; Samarth Life Sciences, Mumbai, India) with CO_2_ (polidocanol and CO_2_ were in a 1:4 ratio) between the 2 syringes. Polidocanol and CO_2_ were mixed back and forth at least 20 times between the 2 syringes. Foam injection was started from the proximal rectum to the distal rectum. The anal canal inferior to the pectinate line is sensitive to pain because of somatic innervation. Caution was exercised in the anal canal near the pectinate line. One to 2 mL of foam was injected per puncture. A total of 12 mL of the sclerosant was injected at each session (Figure 2). Hematochezia markedly improved after 3 sessions of sclerotherapy performed at 2-week intervals. The patient was followed on a laxative and hematinic.

Nine-month follow-up showed occasional rectal bleeding, stable hemoglobin (12.0 g/dL), and a remarkable improvement in MRI and colonoscopy images. Follow-up colonoscopy showed remarkable sclerosis of engorged veins (Figure 2). A few residual engorged veins were noted. T2W pelvic MRI images showed reduced rectal wall thickness, a decrease in vascular signals, and an increase in mesorectal fat signal (Figure 2). The fourth session of foam sclerotherapy was performed for residual VMs. The laxative and hematinic were continued. The patient is doing well after 3 years of follow-up.

## DISCUSSION

Treatment of colorectal VMs has been described as a case report or a small series of cases. Methods of treatment include surgery, endoscopic sclerotherapy, and very rarely portal vein sclerotherapy.^4^ Surgery is an option if the lesion can be resected completely, and the anastomosis site is disease-free. Surgical resection with anastomosis is indicated if the anal sphincter is not affected. In properly selected cases, surgery with sphincter preservation is the treatment of choice with low anterior resection or pull-through transection techniques.^4,5^

Because of anal canal and perirectal structure involvement, we planned for endoscopic microfoam sclerotherapy. Endoscopic injection sclerotherapy has been reported as an effective treatment of small colorectal VMs. However, it has rarely been described in the treatment of large VMs.^3–5^ However, sclerotherapy carries risk of complications which include pain, ulcerations, hemorrhage, strictures, pleural effusions, pericarditis, perforation, and very rarely pulmonary embolization. The injection of large volumes of sclerosants in a single session can cause side effects including air embolization. Therefore, multiple sessions are required for larger lesions. Two cases of rectal hemangioma were treated with 13–15 sessions of endoscopic sclerotherapy.^6^

A liquid sclerosant (polidocanol) can be converted into microfoam using CO_2_. Microfoam causes even distribution of the sclerosant over the endothelium, and CO_2_ reduces chances of embolization. Therefore, the required quantity of sclerosants and their side effects can be remarkably reduced. A larger volume of a sclerosant can be injected in one session.^7,8^ It has been used for the treatment of large varicose veins and other vascular malformations.^8^ We required 4 sessions of sclerotherapy.

In conclusion, anorectal VM is a rare disease. Endoscopic foam sclerotherapy seems to be a safe alternative to surgery for the treatment of large anorectal VMs.

## DISCLOSURES

Author contributions: A.K. Jha and A. Suchismita were involved in designing and writing the manuscript. V.M. Dayal assisted in the review of the literature. All authors read and approved the final manuscript. A.K. Jha is the article guarantor.

Financial disclosure: None to report.

Informed consent was obtained for this case report.
